# Reference Interval Determination for Digital Rectal Temperature in Healthy Ferrets (*Mustela putorius furo*)

**DOI:** 10.3390/ani16091388

**Published:** 2026-05-02

**Authors:** Hayley S. Stratton, Elizabeth G. Duke, Ronald Kent Passingham, Tara M. Harrison

**Affiliations:** Department of Clinical Sciences, North Carolina State University College of Veterinary Medicine, Raleigh, NC 27607, USA; hsstratt@ncsu.edu (H.S.S.); ecgraebe@ncsu.edu (E.G.D.); rkpassin@ncsu.edu (R.K.P.)

**Keywords:** rectal temperature, reference interval, ferret, *Mustela putorius furo*

## Abstract

While digital rectal thermometry is commonly used for assessing health and identifying illness in ferrets, there is little evidence to support frequently cited ranges of normal rectal temperature in this species. The objective of this study was to establish a reference interval for digital rectal temperature in healthy ferrets (*Mustela putorius furo*). Rectal temperatures were measured using digital rectal thermometry in 56 healthy ferrets greater than four months of age. The reference interval was established using robust methods for reference interval determination, in adherence with the recommendations of the American Society for Veterinary Clinical Pathology and the Clinical and Laboratory Standards Institute. The reference interval for digital rectal temperature of healthy ferrets greater than four months of age was determined to range from 38.1 °C (confidence interval [CI] = 37.9 to 38.3 °C; 100.6 °F [CI = 100.3 to 100.9 °F]) at the lower limit to 39.9 °C (CI = 39.8 to 40.1 °C; 103.9 °F [CI = 103.6 to 104.2 °F]) at the upper limit. This reference interval for digital rectal temperature (38.1–39.9 °C [100.6–103.9 °F]) will be valuable to clinicians both performing routine health assessments and treating clinically ill ferrets.

## Simple Summary

Rectal temperature is commonly measured by veterinarians to monitor health and identify illness in ferrets. Reference ranges for normal rectal temperature in ferrets have been published, but there is little evidence to support these ranges. The objective of this study was to establish a reference interval for normal rectal temperature in healthy ferrets. Rectal temperatures were measured using a digital rectal thermometer in 56 healthy ferrets greater than four months of age. The reference interval was established using standard statistical methods recommended by the American Society for Veterinary Clinical Pathology and the Clinical and Laboratory Standards Institute. The reference interval for digital rectal temperature of healthy ferrets greater than four months of age was determined to range from 38.1–39.9 °C (100.6–103.9 °F). This information will be valuable to veterinarians both performing wellness examinations on healthy animals and treating ill ferrets.

## 1. Introduction

Mammals are homeothermic, meaning they possess the physiologic ability to maintain a constant core body temperature, regardless of fluctuations in external environmental temperature [1,2]. Body temperature is frequently measured to assess the health status of ferrets, as an abnormally low or high body temperature may be an indication of clinical disease [3]. A decrease in body temperature, or hypothermia, can be classified as primary or secondary [1,4,5]. Primary hypothermia occurs following prolonged exposure to a cold environment, whereas secondary hypothermia occurs when illness, injury, or administration of drugs alters an animal’s ability to thermoregulate and generate heat [1,4,5]. In contrast, hyperthermia refers to an elevation in core body temperature above normal. Hyperthermia may be the result of pyrexia, where the anterior hypothalamus increases the body’s set point temperature [1,6]. Hyperthermia can also result from physiologic, pathologic, or pharmacologic changes that cause the body to accumulate more heat than it loses [1,6].

As most physiologic processes within the body are optimized at a normal body temperature, deviations from normothermia can have significant negative impacts on numerous organ systems, including the cardiovascular, gastrointestinal, hepatic, nervous, neuromuscular, renal, and respiratory systems [1,4,5,6,7,8]. Additionally, abnormal body temperature may lead to deleterious effects on acid–base balance, coagulation, and electrolyte homeostasis [1,4,5,6,7,8].

Ferrets, like other small mammals, are prone to developing hypothermia due to their small size and increased surface area to body weight ratio [9,10]. Additionally, hypothermia has previously been demonstrated to be a significant negative prognostic indicator in ferrets [1]. In a retrospective study in client-owned ferrets, the odds of death for hypothermic ferrets (<37.8 °C, 100.0 °F) were 3.72 times the odds of death for normothermic ferrets (37.8–40 °C, 100.0–104.0 °F) [1]. For every 0.56 °C (1.0 °F) below 37.8 °C (100.0 °F), the odds of death increased 1.49 times [1]. Small mammals, including ferrets, frequently develop hypothermia in early decompensatory stages of shock; due to their small size, ferrets are prone to developing dehydration and subsequently hypovolemic shock due to decreased water intake or excessive fluid losses [1,3].

In ferrets, hyperthermia due to pyrexia can occur secondary to numerous disease processes. Major etiologies of differential diagnoses for pyrexia include infectious, non-infectious inflammatory, and neoplastic diseases [11]. Selected differentials of importance for pyrexia in ferrets include sepsis [3], influenza virus [12], canine distemper virus [13], ferret systemic coronaviral disease [14], and disseminated idiopathic myofasciitis (DIM) [15]. Ferrets are also vulnerable to hyperthermia secondary to heat stress and heatstroke [3,16]. Ferrets lack sweat glands, and their high surface-area-to-body-weight ratio increases their risk of heat absorption in hot environments [3,17].

Body temperature measurement via digital rectal thermometry is commonly performed in ferrets during routine health examinations and monitoring of clinically ill patients [3,18]. As many of the aforementioned disease processes that cause derangements in body temperature can have non-specific clinical signs, it is important to perform thermometry in any ferrets showing signs of illness [1,3,11,12,13,14,15,16]. The normal rectal temperature of healthy ferrets has been reported to range from 37.8–38.1 °C (100.0–100.5 °F) at the low end to 39.2–40.0 °C (102.5–104.0 °F) at the high end [18,19,20,21,22,23,24,25]; however, the methods by which these ranges were determined are not well described. The objective of this study was to establish a reference interval for digital rectal temperature in healthy ferrets greater than four months of age.

## 2. Materials and Methods

### 2.1. Animals

The study protocol was approved by the Institutional Animal Care and Use Committee of the North Carolina State University (NCSU) College of Veterinary Medicine (IACUC #25-293). Fifty-six ferrets (*Mustela putorius furo*; *n* = 27 females and *n* = 29 males) were evaluated, ranging in age from four months to an estimated age of 6.5 years. Animals ranged in weight from 0.64 to 2.27 kg with body condition score ranging from 4/9 to 6/9. Eleven ferrets were considered juveniles (less than 1 year of age; *n* = 3 females and *n* = 8 males), and 44 ferrets were considered adults (1 year of age or older; *n* = 24 females and *n* = 20 males) at the time of the study [26]; an age was not provided for one ferret. Eight male ferrets (*n* = 7 juveniles and *n* = 1 adult) and four female ferrets (*n* = 1 juvenile and *n* = 3 adults) were intact. The remaining ferrets were neutered. None of the intact female ferrets were pregnant at the time of inclusion in the study. The ferrets were part of several physical examination and surgical teaching laboratories offered to veterinary students at the NCSU College of Veterinary Medicine between October 2023 and October 2025. The majority of ferrets (*n* = 39) came from a local ferret rescue, while a subset were ferrets obtained from a commercial breeding facility (*n* = 12; Marshall BioResources, North Rose, NY, USA) and ferrets owned by a local Association of Zoos and Aquariums (AZA)-accredited facility (*n* = 5). Ferrets were housed in a climate-controlled environment (18–21 °C [64.4–69.8 °F]) for at least one hour prior to the start of this study. All ferrets had free access to a pelleted diet for at least one hour prior to the start of the study, and ferrets were offered small amounts of food during examinations to facilitate handling.

In adherence with the American Society for Veterinary Clinical Pathology (ASVCP) reference interval guidelines, health status was defined by history and physical examination performed at the time of data collection [27]. Animals were included in this study if they did not show signs of clinical illness in the two weeks preceding data collection and if they did not have physical examination abnormalities considered likely by a veterinarian to be related to a disease process that would cause a deviation in rectal temperature. Clinicopathologic analysis was not performed. All ferrets were determined to be clinically healthy based on the inclusion criteria. No animals were reported to show clinical signs of illness in the two weeks preceding data collection. Twenty-nine animals had no abnormalities appreciated on physical examination, while 27 animals had one or more abnormalities noted on physical examination. Abnormal physical examination findings are reported as follows: dental disease (*n* = 18), cutaneous lesions consistent in appearance to cutaneous mast cell tumors (*n* = 5), hair loss (*n* = 3), fleas (*n* = 2), seborrhea (*n* = 2), unilateral cataract (*n* = 1), chronic auricular hematoma (*n* = 1), unilateral enucleation (*n* = 1), mild unilateral serous nasal discharge (*n* = 1), a historically fractured digit (*n* = 1), and a cytologically diagnosed benign cutaneous cyst (*n* = 1). Consent for inclusion in this study was obtained from the ferrets’ organizations by a representative of the NCSU College of Veterinary Medicine.

### 2.2. Data Collection

Rectal temperatures were measured for all animals using a digital rectal thermometer (Vicks SpeedRead Digital Thermometer; Procter & Gamble, Cincinnati, OH, USA). The digital thermometer had a temperature range of 32.0 °C to 42.9 °C (89.6–109.2 °F) and a manufacturer-reported accuracy of ±0.1 °C (±0.2 °F). The digital thermometer displayed temperature values in less than 15 s. This device was originally calibrated by the manufacturer but was not recalibrated prior to utilization in this study, as the temperature range of the thermometer did not allow for use of the gold-standard thermometer calibration methods of immersion in a circulating hot-water bath (100 °C [212 °F]) or immersion in an ice-water bath (0 °C [32 °F]); thus, it was assumed that the thermometer was performing within the manufacturer’s published specifications.

A single rectal temperature measurement was collected from each ferret included in this study by a veterinarian. Ferrets were manually restrained, and the thermometer was lubricated with a water-soluble lubricating jelly and inserted into the rectum to a depth of approximately 2 cm until an audible beep signal indicated the peak temperature had been reached. No ferrets in the study had evidence of prostatic enlargement that would hinder rectal temperature measurement.

Following completion of the study, the thermometer was verified to be performing within the manufacturer’s published specifications. The study thermometer was placed in a water bath with a temperature set at 37.8 °C (100 °F). The temperature of the water bath was confirmed using a different thermometer that was calibrated via immersion in an ice-water bath (0 °C [32 °F]; ThermoPro TP03H; ThermoPro, Toronto, ON, Canada). Five readings were obtained with the study thermometer, which were between 37.8–37.9 °C (100.0–100.2 °F). Based on this, the study thermometer was confirmed to be performing within the manufacturer’s reported accuracy of ±0.1 °C (±0.2 °F).

### 2.3. Statistical Analysis

Normality was assessed with a Shapiro–Wilk test, and continuous variables are summarized as mean and standard deviation or median and interquartile range based on their distribution. A value of *p* ≤ 0.05 was considered statistically significant. Individual ferrets with abnormal physical examination findings were categorized as having mild, moderate, or severe physical examination abnormalities, based on the likelihood that these abnormalities could contribute to systemic disease. No ferrets were categorized as having severe physical examination abnormalities. Ferrets with dental disease (*n* = 18) were categorized as having moderate physical examination abnormalities due to the potential for dental-associated infection; however, no evidence of dental-associated infection was appreciated on physical examination in any ferrets. All other ferrets with abnormalities were categorized as having mild physical examination abnormalities. As only nine animals had mild physical examination abnormalities, these animals were grouped with ferrets that had no physical examination abnormalities for analysis.

Unpaired *t* tests were performed to assess for differences in rectal temperature between sexes, between neuter status within sexes, and between physical examination findings. A Mann–Whitney U test was used to assess for a difference in rectal temperature between age classes. Additionally, a general linear model was utilized to evaluate the effects of sex, neuter status, physical examination abnormalities, and their interactions on rectal temperature. Age was initially considered but excluded from the general linear model as the residuals were non-normally distributed.

The reference interval was established using robust methods for reference interval determination, in adherence with the recommendations of the ASVCP and the Clinical and Laboratory Standards Institute [27]. All analyses were performed using statistical software (Microsoft Excel 365, Microsoft Corporation; SPSS Statistics version 31, IBM; MedCalc Statistical Software version 23.3.7, MedCalc Software).

## 3. Results

The median age and interquartile range for all ferrets were 2 years and 1–3 years, respectively.

Digital rectal temperatures were normally distributed (*p* = 0.76). There was no significant difference in rectal temperature between sexes (*p* = 0.95); within sexes, there was no significant difference in rectal temperature between neutered and intact animals (*p* = 0.12 and *p* = 0.66 for females and males, respectively). There was also no significant difference in rectal temperature between juvenile and adult ferrets (*p* = 0.14). Additionally, there was no significant difference in rectal temperature between ferrets with no or mild physical examination abnormalities and ferrets with moderate physical examination abnormalities (*p* = 0.99). The general linear model did not reveal a significant effect of sex, neuter status, or physical examination findings on rectal temperature (*p* = 0.52, 0.18, and 0.88, respectively). There were also no significant interactions between any of the variables (*p* > 0.05 for all comparisons).

**Figure 1:**
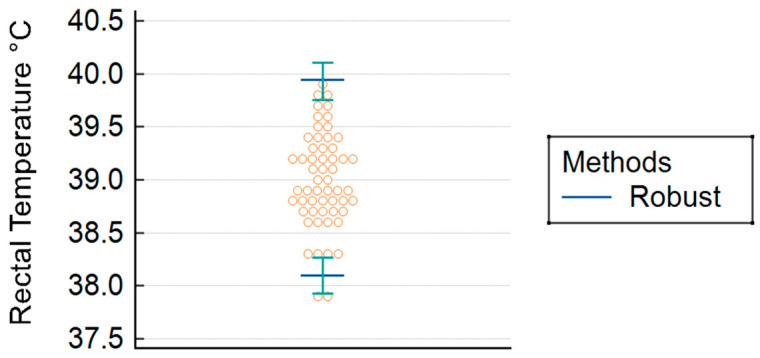
Distribution of rectal temperatures in 56 healthy ferrets greater than four months of age. Bracketed lines represent the upper and lower reference limits and 90% confidence intervals, calculated using robust methods for reference interval determination. The reference interval was determined to range from 38.1 °C (90% confidence interval [CI] = 37.9 to 38.3 °C; 100.6 °F [90% CI = 100.3 to 100.9 °F]) at the lower limit to 39.9 °C (90% CI = 39.8 to 40.1 °C; 103.9 °F [90% CI = 103.6 to 104.2 °F]) at the upper limit.

The mean ± standard deviation of rectal temperature was 39.0 ± 0.46 °C (102.2 ± 0.82 °F). The reference interval for digital rectal temperature of healthy ferrets greater than four months of age was determined to range from 38.1 °C (90% confidence interval [CI] = 37.9 to 38.3 °C; 100.6 °F [90% CI = 100.3 to 100.9 °F]) at the lower limit to 39.9 °C (90% CI = 39.8 to 40.1 °C; 103.9 °F [90% CI = 103.6 to 104.2 °F]) at the upper limit (Figure 1).

## 4. Discussion

The normal range of digital rectal temperature in healthy ferrets greater than four months of age was determined to be 38.1–39.9 °C (100.6–103.9 °F). Multiple sources cite a normal temperature range of 37.8–40.0 °C (100.0–104.0 °F) [18,19,22,23,25], with single sources citing normal temperature ranges of 37.8–39.4 °C (100.0–103.0 °F) [24] and 38.1–39.2 °C (100.5–102.5 °F) [20]; however, the methods and conditions under which these ranges were determined are not documented. Comparatively, the reference interval for rectal temperature established in this study is similar to the most commonly published range of 37.8–40.0 °C (100.0–104.0 °F) [18,19,22,23,25].

Three prior studies reported ranges of digital rectal temperature of ferrets as 36.7–39.4 °C (mean 38.3 °C; 98.0–103.0 °F, mean 101.0 °F) [28], 37.9–39.6 °C (mean 38.8 °C; 100.2–103.3 °F, mean 101.8 °F) [29], and 37.2–39.2 °C (mean 38.2 °C; 99.0–102.6 °F, mean 100.7 °F) [30]; however, these studies utilized a small population of ferrets (*n* = 20, *n* = 27, and *n* = 16, respectively) that precluded reference interval determination. The upper limit of the reference interval determined in this study is higher than the upper limit of these previously reported ranges [28,29,30]. This difference may be a result of the more robust sample size utilized in this study. Alternatively, these differences could be a result of differences in instrumentation or sample population. For example, Maxwell et al. utilized a small population of purpose-bred laboratory ferrets between five and six months of age, whereas Aguilar et al. utilized a small population of adult ferrets that presented to a veterinary hospital for routine preventative care [29,30]. The results of this study suggest that the use of these previously published ranges by clinicians could lead to an overdiagnosis of mild pyrexia; therefore, clinicians should exercise clinical judgment when interpreting rectal temperature measurements in ferrets.

Body temperature measurement is considered a routine component of wellness examinations in ferrets [20]. Additionally, multiple disease processes have been associated with alterations in body temperature in ferrets [3,11,12,13,14,15,16]. Furthermore, hypothermia has previously been identified as a significant negative prognostic indicator in ferrets [1]. Given both the diagnostic and prognostic relevance of body temperature, thermometry should be performed in all ferrets presenting with clinical signs of illness.

A previous study demonstrated that tympanic thermometry on the dorsal skin may be a suitable alternative to digital rectal thermometry in healthy ferrets [28]. The use of tympanic thermometry on the dorsal skin is less invasive than rectal thermometry and may reduce discomfort, stress, and the risk of mucosal irritation in ferrets. While tympanic thermometry on the dorsal skin had the lowest mean difference when compared with rectal temperature measurement, the authors of the present study have appreciated clinically significant differences between tympanic thermometry on the dorsal skin and rectal thermometry in ferrets with severe hypothermia and hyperthermia [28]; therefore, while tympanic thermometry on the dorsal skin may be a suitable alternative to rectal thermometry in clinically healthy animals, rectal thermometry should still be considered in ferrets with signs of clinical disease. Additionally, abnormal temperature readings obtained by tympanic thermometry on the dorsal skin should be verified via rectal thermometry.

The same study that evaluated tympanic thermometry on the dorsal skin of healthy ferrets also evaluated tympanic thermometry on the ear, inguinal, axillary, and noncontact infrared thermometry [28]. Compared to rectal temperature measurement, all four of these temperature measurement methods had a mean difference that was greater in magnitude than 0.5 °C, which exceeds commonly used mean difference thresholds [28]. In all cases, the mean difference in temperature measurement was lower compared to rectal temperature [28]. These findings further support the use of rectal thermometry for temperature measurement, particularly in clinically ill ferrets.

A limitation to this study was that restraint was performed by multiple veterinary students, as data was collected during student teaching laboratories. Additionally, while rectal temperatures were performed as quickly as possible after the start of handling, the time between start of handling and rectal temperature measurement was not standardized across all ferrets. The lack of standardized handling protocols could be a source of preanalytical variability in temperature measurements. While some ferrets tolerated rectal thermometry well, other ferrets showed clinical signs of stress including attempting to pull away from the thermometer, vocalization, urination, and defecation. Stress-induced hyperthermia has previously been reported in ferrets and cannot be excluded as a possibility in this population, particularly as handling time was not standardized between ferrets prior to temperature measurement [20,31]. If stress-induced hyperthermia occurred within the study population, this may have artificially increased the upper limit of the reference interval.

Veterinarians should therefore exercise clinical judgment when interpreting rectal temperature measurements in ferrets, as a measurement at the upper end of the reported reference interval in an animal that is very calm or showing signs of clinical illness could still represent an increase compared to the animal’s normal baseline temperature. While stress-induced increases in body temperature have been reported in ferrets, a recent study reported consistency between pre- and post-handling temperature measurements in ferrets [28]; therefore, the effects of handling on rectal temperature measurement in this study population are not known. Future studies should aim to minimize and standardize handling time prior to rectal temperature measurement to reduce this source of preanalytical variability.

Additionally, the ferrets included in this study were determined to be clinically healthy as they were not reported to show clinical signs of illness in the two weeks preceding data collection; however, physical examination performed at the time of the study revealed numerous clinical findings. The most common clinical findings identified in this population were dental disease and cutaneous lesions consistent in appearance with mast cell tumors. Based on clinical experience, the authors considered it unlikely that the findings identified on physical examination were associated with a disease process that would cause a deviation in rectal temperature from the animal’s baseline. Additionally, significance testing determined that there was no significant difference in rectal temperature between ferrets with no or mild physical examination abnormalities and ferrets with moderate physical examination abnormalities; therefore, these animals were included in the study population. However, the authors cannot definitively exclude the possibility that these clinical findings were related to a disease process that would alter rectal temperature. Additionally, clinicopathologic analysis, such as blood work, was not performed on the study population, so it is also possible that these animals could have had subclinical illness that may have impacted rectal temperature measurements.

Another limitation of this study was that the digital rectal thermometer was not recalibrated to ensure its accuracy prior to use in this study. Many commercially available medical-grade digital rectal thermometers, including the one used in this study, are factory-calibrated for accuracy and do not require user calibration prior to use. In this study, it was assumed that the thermometer was calibrated correctly by the manufacturer and performing within the manufacturer’s published specifications; however, this assumption was not verified prior to completion of the study, and this could be a potential source of measurement error in the unlikely event that the thermometer was calibrated incorrectly by the manufacturer. Following completion of the study, the thermometer was verified to be performing within the manufacturer’s reported accuracy of ±0.1 °C (±0.2 °F). Future studies should utilize a digital rectal thermometer that includes a function for users to verify the calibration of the unit.

Finally, this study utilized a heterogenous population of ferrets, combining data from both sexes and from a wide age distribution. Statistical analysis confirmed that there was no statistical difference in temperature measurements between male and female ferrets or between juvenile and adult ferrets, so data from all ferrets were combined for further analysis. Ferret kits are altricial and rely on the jill for homeostasis for the first three weeks of life [26]. The youngest animal included in this study was four months of age; thus, all animals included in this study were presumed capable of thermoregulation, and the lack of significant difference in temperature between the juvenile and adult animals included in this study was not unexpected. While no significant differences in rectal temperature between sexes or neuter statuses were identified in this study, it is possible that the sample size was too small to detect significant differences between groups. Prior studies in cats have identified a lower rectal temperature in pregnant cats compared to nonpregnant cats [32,33]. While these findings are specific to cats, the effects of pregnancy status on rectal temperature warrants further investigation in other species, including ferrets. Further studies should utilize a larger population of ferrets to allow sex-based, age-based, and reproductive status-based subgroup analysis of rectal temperatures.

## 5. Conclusions

This study provides a reference interval for digital rectal temperature in healthy ferrets greater than four months of age (38.1–39.9 °C [100.6–103.9 °F]). This information will be valuable to clinicians both performing routine health assessments and treating clinically ill ferrets. As many disease processes that cause derangements in body temperature in ferrets can have non-specific clinical signs, it is important to perform thermometry on any ferrets showing signs of illness. Further studies should utilize a larger population of ferrets to allow determination of digital rectal temperature reference intervals for sex-based, age-based, and reproductive status-based subgroups.

## Data Availability

The raw data supporting the conclusions of this article will be made available by the authors on request.
